# Prevalence and correlates of anxiety and depression among chronically ill older adults in Zunyi, China: a cross-sectional study

**DOI:** 10.3389/fpsyg.2025.1560650

**Published:** 2025-04-03

**Authors:** Xiaoling Zhao, Xiaoli Yuan, Dan Meng, Heting Liang, Yan Xiong, Yunting Li, Shuang Li, Mei He, Pan Cai

**Affiliations:** ^1^Department of Nursing, Affiliated Hospital of Zunyi Medical University, Zunyi, Guizhou, China; ^2^The Third Affiliated Hospital of Zunyi Medical University/The First People's Hospital of Zunyi, Zunyi, Guizhou, China; ^3^School of Nursing, Zunyi Medical University, Zunyi, Guizhou, China

**Keywords:** chronically ill elderly in Zunyi, China, anxiety, depression, prevalence, influencing factors

## Abstract

**Background:**

Against the background of an aging population, the prevalence of mental health problems among elderly people with chronic diseases is steadily increasing. The prevalence of mental health problems is higher in less economically developed regions, but there are still limited reports on the mental health data of elderly people with chronic diseases in less developed western regions.

**Objective:**

To find out the prevalence of anxiety and depression among chronically ill elderly people in Zunyi, China, and to analyze the risk factors for the prevalence of anxiety and depression.

**Methods:**

A stratified randomized whole-cluster sampling method with face-to-face questionnaires was used to survey people aged 60 years and older with chronic diseases in Zunyi, China, from March 2022 to October 2023. Relevant demographic and clinical data were collected using structured questionnaire contents of sociodemographic characteristics, Self-Assessment Scale for Anxiety (SAS), Geriatric Depression Scale (GDS-15), Brief Miniature Nutritional Assessment Scale (MNA-SF), Pittsburgh Sleep Quality Index (PSQI), Barthel Index (BI), etc., and bivariate analyses were carried out to explore the anxiety according to the different characteristics of the subjects Bivariate analyses were conducted to explore the differences in anxiety and depression according to the different characteristics of the subjects, and multifactorial logistic regression analyses were conducted to test the factors affecting anxiety and depression.

**Findings:**

A total of 9,998 subjects were enrolled in this study, and 9,821 valid questionnaires were obtained. The prevalence of anxiety was 26.83% (*95% CI*: 26.00–27.70), the prevalence of depression was 10.33% (*95% CI*: 9.70–10.90), and the prevalence of anxiety combined with depression was 6.88% (95% CI: 6.40–7.40). The chronic diseases included in this study encompass cardiovascular diseases, endocrine system diseases, cerebrovascular diseases, degenerative diseases, respiratory system diseases, digestive system diseases, kidney diseases, and cancer. Compared to patients with other chronic diseases, those suffering from cardiovascular diseases exhibit a higher prevalence of anxiety, depression, and the co-occurrence of both anxiety and depression. The results of multifactorial logistic regression analysis showed that advanced age, high literacy, high average monthly household income, regular physical exercise, adequate material and emotional support, and mild dependence in daily life were protective factors for depression among chronically ill older adults (*P* < 0.05). Living in rural areas, moderate ability to perform activities of daily living, heavy dependence, poor self-assessed health, presence of malnutrition, presence of debilitation and in the pre-debilitating stage, and presence of sleep disorders were independent risk factors for depression in chronically ill older adults (*P* < 0.05). High literacy, high average monthly family income, regular physical exercise, regular social activities, sufficient material and emotional support, living in a nursing facility, and mild dependence in daily life were protective factors for anxiety in chronically ill older adults (*P* < 0.05). Advanced age, childlessness, moderate ability to perform activities of daily living, heavy dependence, poor self-assessed health, the presence of malnutrition, the presence of infirmity and pre-infirmity, and the presence of sleep disorders were independent risk factors for anxiety in chronically ill older adults (*P* < 0.05).

**Interpretation:**

The prevalence of anxiety among elderly individuals with chronic diseases in Zunyi City, China, is higher than the global average, whereas the prevalence of depression is lower than the global level. Patients with cardiovascular diseases exhibit a higher prevalence of anxiety, depression, and the co-occurrence of both anxiety and depression. Factors such as economic income, education level, lifestyle habits, and medical history influence the occurrence of anxiety and depression in elderly individuals with chronic diseases. This highlights the need to promote screening for anxiety and depression among the elderly with chronic conditions and to enhance community awareness of mental health issues in the elderly population.

## 1 Introduction

Against the backdrop of an aging population, the prevalence of chronic diseases among the elderly is continuously rising, posing a significant challenge to healthcare systems worldwide (Su et al., 2024). Relevant data indicate that ~180 million elderly individuals globally currently suffer from chronic diseases, with over 75% of the elderly diagnosed with one or more chronic conditions. Chronic diseases account for 71% of all global deaths annually (Li et al., 2024). Chronic diseases not only diminish the quality of life but also lead to health emergencies, severe complications, and even death (Sharma et al., 2021). Moreover, the cost of treating chronic diseases increases exponentially with the number of conditions, placing a substantial financial burden on healthcare systems and patients' families (Hong et al., 2022).

Chronic-disease-related depression and anxiety are prevalent mental health issues in contemporary society, with their prevalence rates steadily rising (Wang Z. et al., 2019). In foreign regions, the prevalence of depression among the elderly ranges from 11.4% to 43% (Zenebe et al., 2021; Behera et al., 2016), and the prevalence of anxiety ranges from 14.2% to 39.4% (Yan et al., 2022; Mutepfa et al., 2021; Cho et al., 2021). Specifically, in Portugal, the prevalence of anxiety among the elderly is 9.6%, and the prevalence of depression is 11.8% (de Sousa et al., 2017). In India, the prevalence rates of anxiety and depression among patients with chronic diseases reach 51.1% and 58.8%, respectively (Swathi, Manjusha, Vadakkiniath and et al., 2023). In China, the prevalence of anxiety and depression among the elderly is 27.0%−37.3% and 11.8%−40.3%, respectively (Lu et al., 2023; Yunming et al., 2012; Sarokhani et al., 2018; Qiao et al., 2024). Specifically, in Hunan Province, China, the prevalence rates of anxiety and depression are 32.74% and 37.34%, respectively (Lu et al., 2023), and the prevalence rates of depressive and anxiety symptoms among the elderly in Shenzhen communities are 10.4% and 11.3%, respectively (Wang et al., 2023). Among the elderly, the long-term, recurrent treatment process of chronic diseases and the likelihood of complications cause patients to suffer from physical pain for extended periods, leading to the emergence of depressive and anxiety symptoms (Zhao et al., 2020). This places a heavy burden on both individuals and society as a whole. Meanwhile, the “Healthy China 2030” Plan Outline clearly states that it is necessary to strengthen the intervention of common mental and psychological problems such as depression and anxiety, and emphasizes the importance of focusing on rural areas and grassroots levels to strengthen the early detection and timely intervention of mental and psychological problems among key populations.

Zunyi, an important city in Guizhou Province, China, faces challenges like lagging economic development, scarce medical resources and limited mental health services. Elderly chronic disease patients here often lack necessary support, and their economic burdens and social isolation fuel anxiety and depression. Meanwhile, research shows that the incidence of chronic diseases in Zunyi region is as high as 46.98% (Zhong et al., 2018). Common chronic diseases such as hypertension and diabetes bring physical discomfort, lifestyle changes, and economic pressure due to long-term illness, making the elderly more prone to anxiety and depression. In addition, Zunyi is located in the southwest region of China and has a unique regional culture and lifestyle, which may have an impact on the mental health of the elderly. For example, local factors such as tea-drinking, alcohol-consuming, mahjong-playing, and ethnic minority cultures can all play a role.

Currently, there are limited reports on the mental health data of the elderly with chronic diseases in less-developed western regions. Therefore, this study aims to understand the current status of anxiety and depression symptoms among elderly individuals with chronic diseases in Zunyi City and to explore the influencing factors, with the goal of providing targeted recommendations and guidance for developing local health policies and interventions tailored to the unique needs of the aging population. Additionally, by offering data on the prevalence of anxiety and depression in the Zunyi region, this study fills a gap in the literature and enriches research on the mental health of the elderly, both in China and globally.

## 2 Materials and methods

### 2.1 Research subjects

The inclusion criteria were as follows: ① age ≥ 60 years; ② able to communicate normally; ③ able to sign informed consent and participate in the study voluntarily.

Exclusion criteria were as follows: ① People with severe schizophrenia, mania and other mental illnesses; ② Patients with severe hearing, vision and speech disorders.

### 2.2 Sampling method

From March 2022 to October 2023, a stratified random sampling method was adopted. Based on the requirements of the National Key R&D Program for Active Health and Aging Response, and considering multiple factors such as research objectives, actual distribution, data comparability, and resource limitations, medical institutions, elderly care institutions, and home-dwelling elderly people in Zunyi City, Guizhou Province were selected as survey subjects at a ratio of 5:1:3.

In the first stage, the representative areas were divided into three categories: medical institutions, elderly care institutions, and home-dwelling elderly people. Medical institutions included hospitals at all levels in Zunyi City, encompassing district people's hospitals, county people's hospitals, traditional Chinese medicine hospitals, township health centers, and community hospitals. Elderly care facilities included institutions at various levels in Zunyi City and its counties. Home-dwelling elderly individuals included those from various communities and villages across townships.

In the second stage, 10 departments were randomly selected from medical institutions, and all eligible subjects within the survey period were recruited. Ten elderly care institutions were chosen from each level of such institutions, and all eligible elderly people within the survey period were included. Ten residential communities or villages were selected from each community. Considering the possibility of ineligible data or refusal to participate in the survey, 2–3 additional locations in each area were selected as alternate households. All eligible subjects within the survey period were recruited. Ultimately, 5,593 subjects were recruited from medical institutions, 1,153 from elderly care institutions, and 3,175 home-dwelling elderly subjects were recruited.

### 2.3 Sample size calculation

By referring to the literature, the median prevalence of anxiety and depression was taken as ~35.00% (Lu et al., 2023). Based on the 95% confidence interval (CI), where α = 1–0.95 = 0.05, *z*0.0_2_5 = 1.96, *P* = 0.35, and δ = 0.01, the following formula was used to calculate the required minimum sample size, which was 8,740. To ensure a sufficient sample size, the sample size was increased by 10%, and the final sample size was 9,614 individuals.


$$\begin{array}{l} N=\;\frac{Z_{\alpha }^{2}P\left(1-P\right)}{\delta^{2}}=\frac{1.96^{2}\times 0.35\;\times \;0.65}{0.01^{2}}\approx \;8,740 \end{array}$$

### 2.4 Content of the survey

#### 2.4.1 Basic information

Information collection included demographic information such as age, gender, education level, marital status, etc.; lifestyle including smoking history, drinking history, etc.; and history of previous diseases (cerebrovascular disease, headache, diabetes mellitus, heart disease, high blood pressure) and other conditions.

#### 2.4.2 Definition of some relevant factors and specification of measurement of relevant indicators

According to Chinese standards, a body mass index (BMI) of ≥28 kg/m^2^ is defined as obesity. Smoking is defined as smoking at least one cigarette per day and having a smoking history of more than 1 year. For leaf tobacco, one cigarette is ~equal to 1 g. Drinking is defined as drinking spirits at least once a week for more than 6 months. Social activities are defined as self-reported participation in social activities (playing cards, mahjong, etc.) for at least 30 min at least once a week. Exercise is defined as self-reported exercise of at least 30 min at least once a week (farm work, square dancing, walking, running, etc.). Chronic diseases are defined as a history of chronic diseases that have been clearly diagnosed in a Class IIA or higher hospital in the past, or chronic diseases currently receiving drug treatment.

#### 2.4.3 Research tools

Anxiety was assessed using the standardized and validated Chinese version of the Self-Rating Anxiety Scale (SAS) (Zung, 1971). The Chinese version of SAS has a Cronbach's α coefficient of 0.733, as validated (Wang et al., 2024). Depression was measured using the standardized and validated Chinese version of the Geriatric Depression Scale (GDS-15) (Albinski et al., 2011). The GDS-15 is an effective and reliable tool for screening depression in the elderly, with the Chinese version demonstrating a Cronbach's α coefficient of 0.846 (Liu et al., 2013). The frailty assessment was conducted using the FRAIL scale, proposed by the International Association of Nutrition and Aging in 2008 (Abellan et al., 2008). Nutritional status was evaluated using the Short-Form Mini-Nutritional Assessment (MNA-SF) (Rubenstein et al., 2001). Sleep quality was assessed using the Pittsburgh Sleep Quality Index (PSQI) (Buysse et al., 1989). Activities of daily living were measured using the Barthel Index (BI) (Mahoney and Barthel, 1965).

#### 2.4.4 Survey process

The survey team was composed of medical staff from the Affiliated Hospital of Zunyi Medical University, including three graduate students. All surveyors were proficient in both the local Guizhou dialect and Mandarin. Prior to the survey, all personnel underwent unified training at the Affiliated Hospital of Zunyi Medical University, totaling four sessions. Evaluators used standardized instructions, questionnaires, and survey terminology to conduct one-on-one scale assessments and data collection with eligible participants in a face-to-face manner. After the survey, designated personnel were responsible for the collection, organization, and preservation of the day's survey data.

The study was approved by the Ethics Committee of Zunyi Medical University (Ethics Approval Number: KLL-2022-814). The entire research process adhered to ethical principles of voluntariness, informed consent, privacy and confidentiality, and non-maleficence. Before the study began, the research objectives, content, and procedures were explained to the participants and their caregivers. Written informed consent was obtained from the participants or their guardians, and the “Informed Consent Form” was signed before screening commenced. Data were encrypted and stored, used solely for this research project, ensuring no harm to participants, who were informed of their right to withdraw at any time.

### 2.5 Statistical analysis

SPSS29.0 statistical software was used, and the count data, when satisfying normal distribution, were expressed as mean ± standard deviation (x ± s), and the independent samples *t*-test was used for comparison between the two groups, while the *Mannu-Whitney U* test was used for comparison between the two groups when they were not normally distributed. Measurement data were expressed as *n (%)*, and comparisons between groups were made using the chi-square test or *Fisher*'s exact probability method. one-way *logistic* regression was used to analyze potential correlates, and single factors found to be correlated were included in the multifactorial *logistic* regression analysis, and stepwise entry of variables was used to determine the final influences on dementia. *P* < 0.05 was taken as the difference being statistically significant.

## 3 Results

### 3.1 General information

A total of 9,998 subjects were included in this study, and 9,821 valid questionnaires were obtained. The effective recovery rate was 98.23%. A total of 177 questionnaires were invalid due to incomplete information, random responses, logical errors, etc. Of the 9,821 respondents included in the study, the mean age of the subjects was 71.89 ± 7.246 years, ranging from 60 to 98 years old, and the percentages of female and male participants were 51.70% and 48.30%, respectively. Nearly half of the population had less than elementary school education. Most of the respondents were married with multiple children and reported a personal monthly income of <RMB 2,000, 35.26% did not exercise, and 42.13% had little to no socialization.

### 3.2 The prevalence of anxiety and depression among the ethnic people

**Figure 1:**
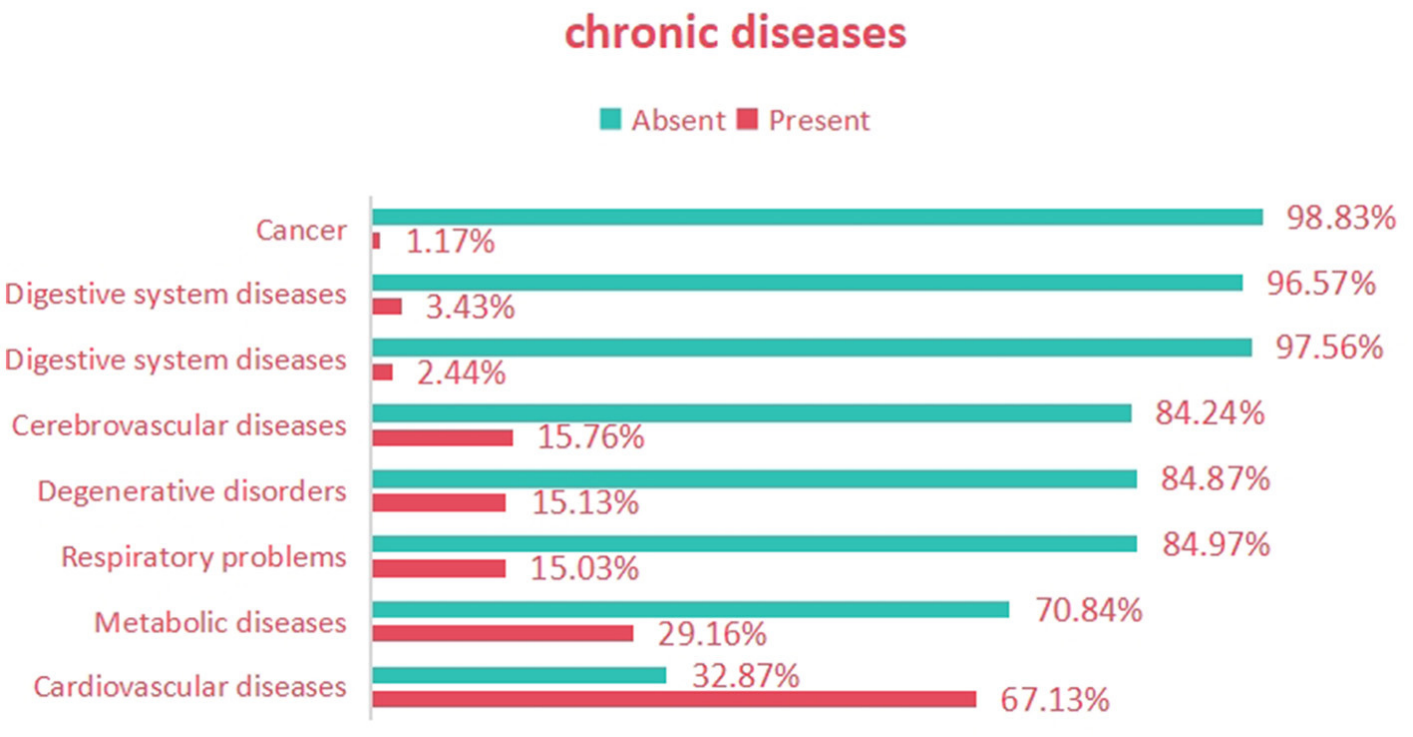
Prevalence of various chronic diseases among participants.

The prevalence of anxiety was 26.83% (95% CI: 25.14–28.52), the prevalence of depression was 10.33% (95% CI: 8.46–12.20), and the prevalence of comorbid anxiety and depression was 6.88% (95% CI: 4.97–8.79). The age group of 71–79 had the highest prevalence. The prevalence was higher in females than in males, and higher in rural areas than in urban areas, as shown in Table 1. Among all participants, the highest proportion of subjects suffered from cardiovascular diseases (67.13%), followed by endocrine system diseases (29.16%), cerebrovascular diseases (15.76%), degenerative diseases (15.13%), respiratory system diseases (15.03%), digestive system diseases (3.43%), kidney diseases (2.44%), and cancer (1.17%), as shown in Figure 1. Compared to patients with other chronic diseases, those with cardiovascular diseases exhibited higher prevalence rates of anxiety, depression, and the co-occurrence of anxiety and depression, as detailed in Table 2.

**Table 1:** Prevalence of anxiety and depression.

**Variable**	**Number (and %) of anxious individuals**	**95% CI**	**Number (and %) of depressed individuals**	**95% CI**	**Number (and %) of individuals with comorbid anxiety and depression**	**95% CI**
Prevalence rate	2,635 (26.83)	25.14–28.52	1,015 (10.33)	8.46–12.20	676 (6.88)	4.97–8.79
**Age/years old**						
60–70	1,034 (39.24)	36.26–42.22	375 (36.95)	32.06–41.84	234 (34.62)	28.52–40.72
71–79	1,159 (43.98)	41.12–46.84	462 (45.52)	40.98–50.06	312 (46.15)	40.62–51.68
Over 80	442 (16.77)	13.29–20.25	178 (17.54)	11.95–23.13	130 (19.23)	12.46–26.01
**Gender**						
Male	1,274 (48.35)	45.61–51.09	497 (48.97)	44.57–53.37	316 (46.75)	41.25–52.25
Female	1,361 (53.65)	51.00–56.30	519 (51.03)	46.73–55.33	360 (53.25)	48.10–58.40
**Residence type**						
Urban	1,291 (48.99)	46.26–51.72	427 (42.07)	37.39–46.75	300 (44.38)	38.76–50.00
Rural	1,344 (51.01)	48.34–53.68	588 (57.93)	53.94–61.92	376 (55.62)	50.60–60.64

**Table 2:** The prevalence of anxiety, depression, and comorbid anxiety and depression among elderly individuals with various chronic diseases.

**Variable**	**Number of People (%)**	**Anxiety (%)**	**Depression (%)**	**Anxiety comorbid with depression (%)**
Cardiovascular diseases	6,593 (67.13)	1,698 (64.44)	652 (64.24)	430 (63.61)
Endocrine system diseases	2,864 (29.16)	760 (28.84)	262 (25.81)	197 (29.14)
Respiratory system diseases	1,476 (15.03)	501 (19.01)	226 (22.27)	159 (23.52)
Degenerative diseases	1,486 (15.13)	506 (19.20)	196 (19.31)	147 (21.75)
Cerebrovascular diseases	1,548 (15.76)	431 (16.36)	236 (23.25)	149 (22.04)
Kidney diseases	240 (2.44)	75 (2.85)	32 (3.15)	26 (3.85)

### 3.3 Analysis of factors influencing anxiety and depression in the elderly population with chronic diseases

Univariate analyses revealed that age, literacy, children's status, type of residence, mode of residence, average monthly household income, type of chronic disease, smoking, participation in social activities, physical activity, social support, ability to perform activities of daily living, self-assessed health status, nutritional status, frailty, and quality of sleep were the factors influencing the anxiety of older adults with chronic disease; age, literacy, children's status, type of residence, mode of residence, average monthly household income, type of chronic disease, participation in social activities, physical activity, social support, self-assessed health status, nutritional status, frailty, and quality of sleep; age, literacy, children's status, type of residence, mode of residence, average monthly family income, type of chronic disease, participation in social activities, physical exercise, social support, ability to perform activities of daily living, self-assessed health status, nutritional status, debilitating conditions, and sleep quality were the influencing factors for depression among older adults with chronic disease; the difference was statistically significant (*P* < 0.05). Details are shown in Table 3.

**Table 3:** Univariate analysis of chronically ill older adults who experienced anxiety and depression.

**Variable (9,821)**	**Anxiety group (2,635) *N* (%)**	***X* ^2^**	***P***	**Depressed group (1,015) *N* (%)**	***X* ^2^**	***P***
**Gender**		10.772	0.001		1.899	0.168
Male	1,283 (48.30)			497 (49.00)		
Female	1,374 (51.70)			518 (51.00)		
**Age/years**		79.351	<0.001		42.425	<0.001
60–70	1,034 (39.22)			375 (36.95)		
71–79	1,159 (43.98)			462 (45.52)		
≥80	442 (16.77)			178 (17.54)		
**Educational attainment**		156.721	<0.001		71.591	<0.001
Below primary school	1,311 (49.75)			522 (51.43)		
Primary school	611 (23.19)			236 (23.25)		
Junior high school	446 (16.92)			170 (16.75)		
Senior high school and above	267 (10.13)			87 (8.57)		
**Marital status**		2.689	0.101		2.070	0.150
Married	2,175 (82.54)			832 (81.97)		
Unmarried/divorced/widowed	460 (17.46)			183 (18.03)		
**Situation of children**		15.793	0.001		23.988	<0.001
Childless	37 (1.40)			12 (1.18)		
1 child	313 (11.88)			103 (10.15)		
2 children	877 (33.28)			303 (29.85)		
3 or more	1,408 (53.43)			597 (58.82)		
**Type of residence**		54.349	<0.001		76.533	<0.001
Urban	1,291 (49.00)			427 (42.07)		
Rural	1,344 (51.00)			588 (57.93)		
**Living pattern**		20.446	<0.001		7.548	0.023
Living with relatives	2,419 (92.33)			929 (91.53)		
Live alone	184 (7.02)			72 (7.09)		
In a nursing home	17 (0.65)			6 (0.59)		
**Average monthly household income, RMB**		78.383	<0.001		16.748	<0.001
<2,000	1,226 (47.45)			375 (37.92)		
2,000–4,000	1,011 (39.13)			474 (47.93)		
>4,000	347 (13.43)			140 (14.16)		
**Types of chronic diseases**		38.018	<0.001		45.199	<0.001
1 type	1,246 (47.29)			441 (43.45)		
2 types	808 (30.66)			315 (31.03)		
3 or more	581 (22.05)			259 (25.52)		
**Smoking**		9.901	0.002		2.545	0.111
No	400 (15.18)			156 (15.37)		
Yes	2,235 (84.82)			859 (84.63)		
**Drinking alcohol**		0.662	0.718		0.388	0.824
Never	1,652 (62.69)			628 (61.87)		
Quit already	683 (25.92)			262 (25.81)		
Yes	300 (11.39)			125 (12.32)		
**Social activity**		142.703	<0.001		114.812	<0.001
No	1,110 (42.13)			525 (51.72)		
1–3 times/week	860 (32.64)			295 (29.06)		
4–6 times/week	353 (13.40)			111 (10.94)		
Everyday	312 (11.84)			84 (8.28)		
**Physical exercise**		429.613	<0.001		314.616	<0.001
No	929 (35.26)			450 (44.33)		
1–3 times/week	799 (30.32)			294 (28.97)		
4–6 times/week	357 (13.55)			98 (9.67)		
Everyday	550 (20.87)			173 (17.04)		
**Social support**		413.316	<0.001		330.292	<0.001
Adequate material and emotional support	2,060 (78.18)			735 (72.41)		
Material support only	108 (4.10)			38 (3.74)		
Emotional support only	141 (5.35)			66 (6.50)		
Lack of material and emotional support	326 (12.37)			176 (17.34)		
**Ability to perform activities of daily living**		319.189	<0.001		758.955	<0.001
Non-dependent	1,172 (44.48)			300 (29.56)		
Mildly dependent	1,030 (39.09)			409 (40.30)		
Moderate dependence	236 (8.96)			146 (14.38)		
Severe dependence	197 (7.48)			160 (15.76)		
**History of surgery**		2.954	0.086		0.737	0.391
Yes	1,120 (42.50)			433 (42.66)		
No	1,515 (57.50)			582 (57.34)		
**Self-assessed health status**		355.488	<0.001		632.001	<0.001
Poor	603 (22.88)			390 (38.42)		
Average	1,771 (67.21)			569 (56.06)		
Good	261 (9.91)			56 (5.52)		
**MNA-SF**		674.972	<0.001		481.137	<0.001
With malnutrition	1,035 (39.28)			490 (48.28)		
Without malnutrition	1,600 (60.72)			525 (51.72)		
**Frailty screening scale**		665.264	<0.001		1,362.827	<0.001
No frailty	910 (34.55)			157 (15.46)		
Pre-weakening	892 (33.85)			273 (26.90)		
Frailty	833 (31.61)			585 (57.64)		
**Pittsburgh Sleep Quality Index**		315.152	<0.001		241.853	<0.001
No sleep disorders	669 (25.39)			175 (17.24)		
With sleep disorder	1,966 (74.61)			840 (82.76)		

There are 218 persons with missing values for average monthly household income.

### 3.4 Multifactorial analysis of anxiety and depression in the chronically ill elderly population

The results of multifactorial *logistic* regression analysis showed that advanced age, high level of education, high average monthly household income, regular physical exercise, sufficient material and emotional support, and mild dependence in daily life were protective factors for the development of depressive illness in chronically ill older adults (*P* < 0.05). Living in rural areas, moderate ability to perform activities of daily living, heavy dependence, poor self-assessed health, presence of malnutrition, presence of debility and in the pre-debilitating stage, and presence of sleep disorders were independent risk factors for depression among chronically ill older adults (*P* < 0.05), and the multifactorial analysis is shown in Table 4. High literacy, high average monthly income of the family, regular physical exercise, frequent participation in social activities, sufficient material and emotional support, living in an elderly care facility, and mild dependence in daily life were protective factors for anxiety in chronically ill older adults (*P* < 0.05). Advanced age, childlessness, moderate ability to perform activities of daily living, heavy dependence, poor self-assessed health status, presence of malnutrition, presence of debility and pre-debilitation, and sleep disorders were independent risk factors for anxiety in older adults with chronic diseases (*P* < 0.05), and the multifactorial analysis is shown in Table 5.

**Table 4:** Multifactorial analysis of depression.

**Variable**	**β**	***SE***	***Wald χ*2**	***P***	***OR (95% CI)***
**Age/years**					
60–70	1.000				
71–79	−0.094	0.087	1.188	0.276	0.910 (0.768–1.078)
≥80	−0.394	0.119	11.042	0.001	0.674 (0.534–0.851)
**Educational attainment**					
Below primary school	1.000				
Primary school	−0.243	0.096	6.352	0.012	0.784 (0.649–0.947)
Junior high school	−0.169	0.115	2.161	0.142	0.844 (0.674–1.058)
Senior high school and above	−0.382	0.155	6.069	0.014	0.683 (0.504–0.925)
**Type of residence**					
Rural	0.315	0.086	13.457	<0.001	1.370 (1.158–1.622)
**Average monthly household income, RMB**					
<2,000	1.000				
2,000–4,000	−0.425	0.089	23.017	<0.001	0.654 (0.549–0.778)
>4,000	−0.620	0.126	0.241	0.623	0.940 (0.734–1.204)
**Physical exercise**					
No	0.403	0.111	13.083	<0.001	1.496 (1.203–1.862)
1–3 times/week	0.449	0.112	16.067	<0.001	1.566 (1.258–1.951)
4–6 times/week	0.220	0.142	2.403	0.121	1.246 (0.944–1.645)
Everyday	1.000				
**Social support**					
Adequate material and emotional support	1.000				
Material support only	0.591	0.210	7.920	0.005	1.805 (1.196–2.725)
Emotional support only	0.817	0.176	21.528	<0.001	2.263 (1.603–3.195)
Lack of material and emotional support	1.146	0.119	93.350	<0.001	3.146 (2.493–3.969)
**Ability to perform activities of daily living**					
Non-dependent	1.000				
Mildly dependent	−0.052	0.095	0.299	0.585	0.949 (0.788–1.144)
Moderate dependence	0.801	0.140	32.896	<0.001	2.229 (1.695–2.931)
Severe dependence	0.989	0.149	44.174	<0.001	2.689 (2.009–3.599)
**Self-assessed health status**					
Poor	1.000				
Average	−0.401	0.093	18.489	<0.001	0.670 (0.558–0.804)
Good	−0.827	0.173	22.822	<0.001	0.437 (0.312–0.614)
**MNA-SF**					
With malnutrition	0.304	0.086	12.641	<0.001	1.355 (1.146–1.603)
Without malnutrition	1.000				
**Frailty screening scale**					
No frailty	1.000				
Pre-weakening	0.757	0.113	44.622	<0.001	2.132 (1.707–2.662)
Frailty	1.977	0.117	285.382	<0.001	7.224 (5.743–9.087)
**Pittsburgh sleep quality index**					
No sleep disorders	1.000				
With sleep disorder	0.760	0.098	60.534	<0.001	2.138 (1.766–2.590)

**Table 5:** Multifactorial analysis of anxiety.

**Variable**	**β**	***SE***	***Wald χ*2**	***P***	***OR (95% CI)***
**Age/years**					
60–70	1.000				
71–79	0.125	0.058	4.708	0.030	1.134 (1.012–1.270)
≥80	0.148	0.083	3.178	0.075	1.160 (0.985–1.365)
**Educational attainment**					
Below primary school	1.000				
Primary school	−0.478	0.065	53.729	<0.001	0.620 (0.546–0.705)
Junior high school	−0.452	0.076	35.310	<0.001	0.636 (0.548–0.738)
Senior high school and above	−0.522	0.098	28.378	<0.001	0.594 (0.490–0.719)
**Average monthly household income, RMB**					
<2,000	1.000				
2,000–4,000	−0.366	0.060	37.176	<0.001	0.694 (0.617–0.780)
>4,000	−0.389	0.083	22.119	<0.001	0.678 (0.577–0.797)
**Childless**					
1 child	1.000				
2 children	−0.593	0.254	5.449	0.020	0.553 (0.336–0.909)
3 or more	−0.487	0.246	3.924	0.048	0.614 (0.379–0.995)
Childless	−0.743	0.245	9.230	0.002	0.476 (0.294–0.768)
**Physical exercise**					
No	0.703	0.081	75.996	<0.001	2.019 (1.724–2.365)
1–3 times/week	0.581	0.082	50.688	<0.001	1.787 (1.523–2.097)
4–6 times/week	0.478	0.104	21.082	<0.001	1.612 (1.315–1.977)
Everyday	1.000				
**Social activity**					
No	1.000				
1–3 times/week	0.435	0.073	35.477	<0.001	1.545 (1.339–1.782)
4–6 times/week	0.238	0.102	5.472	0.019	1.268 (1.039–1.548)
Everyday	0.218	0.091	5.799	0.016	1.244 (1.041–1.486)
**Social support**					
Adequate material and emotional support	1.000				
Material support only	0.927	0.153	36.722	<0.001	2.527 (1.872–3.411)
Emotional support only	0.914	0.142	41.599	<0.001	2.495 (1.890–3.294)
Lack of material and emotional support	0.953	0.097	97.157	<0.001	2.594 (2.146–3.136)
**Living pattern**					
Living with relatives	1.000				
Live alone	0.007	0.107	0.004	0.950	1.007 (0.816–1.242)
In a nursing home	−0.832	0.287	8.387	0.004	0.435 (0.248–0.764)
**Ability to perform activities of daily living**					
Non-dependent	1.000				
Mildly dependent	−0.243	0.061	15.869	<0.001	0.784 (0.695–0.884)
Moderate dependence	0.302	0.114	7.040	0.008	1.352 (1.082–1.690)
Severe dependence	0.119	0.131	0.826	0.363	1.126 (0.872–1.455)
**Self-assessed health status**					
Poor	1.000				
Average	−0.068	0.076	0.809	0.368	0.934 (0.805–1.084)
Good	−0.458	0.105	19.136	<0.001	0.633 (0.515–0.777)
**MNA-SF**					
With malnutrition	0.749	0.062	148.108	<0.001	2.115 (1.875-2.386)
Without malnutrition	1.000				
**Frailty screening scale**					
No frailty	1.000				
Pre-weakening	0.357	0.063	31.663	<0.001	1.429 (1.262–1.618)
Frailty	0.777	0.080	94.942	<0.001	2.176 (1.861-2.544)
**Pittsburgh sleep quality index**					
No sleep disorders	1.000				
With sleep disorder	0.638	0.058	121.365	<0.001	1.893 (1.690-2.121)

## 4 Discussion

This study aims to investigate the prevalence and associated factors of anxiety and depression among patients with various chronic diseases. The specific chronic diseases examined include cardiovascular diseases, endocrine system diseases, cerebrovascular diseases, degenerative diseases, respiratory system diseases, digestive system diseases, kidney diseases, and cancer. Among these chronic conditions, cardiovascular diseases had the highest prevalence. This study underscores the importance of addressing mental health issues in the context of chronic diseases. It not only provides data support for understanding the mental health status of elderly patients with chronic diseases but also offers a reference for formulating public health strategies tailored to local realities, particularly in terms of resource allocation and mental health interventions. Furthermore, by drawing on the experiences from the Zunyi region, this study can provide insights for public health policies in other underdeveloped areas, promoting the establishment of a more comprehensive mental health service system for the elderly.

### 4.1 Prevalence of anxiety and depression in the elderly population with chronic diseases in Zunyi City

This study investigated the prevalence of depression and anxiety among elderly patients with chronic diseases and found that the prevalence rates of anxiety and depression among the elderly were 26.83% and 10.33%, respectively. This study showed that the prevalence of anxiety was higher than the global level (16.5%), while the prevalence of depression was lower than the global level (19.2%) (Jalali et al., 2024). Compared with the results of surveys on elderly populations in foreign countries, the prevalence rates of anxiety and depression in this study were both lower than those of the elderly in Myanmar (39.4% for anxiety and 35.6% for depression) (Cho et al., 2021). When compared with various regions in Europe and Africa, the prevalence of anxiety in this study was higher than that of the elderly in those regions (14.1%−20.8%), but the prevalence of depression was much lower than that of the elderly in those regions (32.2%−47.1%) (Mutepfa et al., 2021; Andreas et al., 2017). Compared with the results of domestic surveys on elderly populations, the prevalence of anxiety in this study was similar to that in a study on community-dwelling elderly in Hunan Province (32.74%) (Lu et al., 2023). The prevalence of depression in this study was similar to that in a study on community-dwelling elderly in Shenzhen, China (10.4%) (Peng et al., 2024), but lower than that of the elderly in Xi'an (27.0%) (Yunming et al., 2012). The differences in prevalence rates are due to factors such as the assessment tools used, the economic development levels of different regions, regional characteristics, demographic compositions, and social and cultural environments.

Firstly, in terms of the long-term management and treatment of chronic diseases, cardiovascular diseases, which are the most common among chronic diseases, can lead to a decline in patients' physical functions. Patients need to take long-term medications and visit doctors regularly. They often worry about the deterioration of their condition, sudden death, or a decline in their quality of life. This uncertainty and worry about the prognosis of the disease can bring huge psychological pressure to patients, making them prone to anxiety and depression (Lopes and Kamau-Mitchell, 2024). At the same time, patients with cardiovascular diseases often have elevated cortisol levels, which may induce anxiety and depression by affecting the hypothalamic-pituitary-adrenal (HPA) axis (Leenen et al., 2017). A study based on 71,214 participants found that anxiety and depression accelerated the formation of cardiometabolic risk factors (such as hypertension and diabetes) and significantly increased the risk of cardiovascular diseases. Depression and anxiety increased the risk of major cardiovascular events such as heart attacks or strokes by ~35% (Civieri et al., 2024). Secondly, the accessibility of healthcare is also a key factor. Zunyi is a region with relatively underdeveloped economy and limited medical resources, facing problems such as uneven distribution of medical resources and insufficient professional medical services. For example, the medical resources in Zunyi City are mainly concentrated in urban areas, and elderly people in rural areas have difficulty in obtaining timely mental health services, which intensifies their concerns about their health conditions and thus increases the prevalence of anxiety. In contrast, the prevalence of depression is lower than the global average, which may be related to the family structure and support system in Chinese society. In China, families are usually the main source of emotional support and daily care for the elderly. This close family bond may alleviate the elderly's depressive emotions to a certain extent. In addition, support programs from communities and the government may also provide a certain amount of psychological and social support for the elderly, thereby reducing the risk of depression.

### 4.2 Age is associated with anxiety and depression

In this study, advanced age was identified as a protective factor against depression. Research has shown that the prevalence of depression decreases with age (Wade and Cairney, 1997). Some studies indicate that older age is associated with better mental health, characterized by lower levels of stress, worry, and negative affect, as well as higher levels of positive affect (Fields et al., 2022). Typically, increasing age is linked to markers of improved mental health, including factors that can reduce depression levels (Blazer, 2003). Some studies suggest that this may be because older adults tend to identify their health issues earlier but face challenges such as limited treatment options and access to information, leading to prolonged periods without medication or regular check-ups. This, in turn, may reduce their focus on disease progression and subsequently lower the risk of mental health problems (Long et al., 2024). However, in this study, age was found to be a risk factor for anxiety, possibly because aging impairs perception, cognitive abilities, verbal expression, comprehension, help-seeking behavior, and disrupts treatment collaboration and adherence (Zhi et al., 2024).

### 4.3 The correlation between educational level, economic status, type of residence, social support, living arrangements, and anxiety and depression

In this study, half of the older adults had less than elementary school, and a lower level of education (less than elementary school) is one of the risk factors for anxiety and depression. A study from France showed that people with a lower level of education have a higher risk of anxiety and depression (Joannès et al., 2023). One of the reasons for this is that the level of education can lead to a lack of health knowledge and health concepts among the elderly, which in turn affects the health care behaviors of elderly people in compliance. At the same time, people with low education level are more likely to suffer from the pressure of economic difficulties, which makes it difficult to get a good job, thus affecting their mental health. Furthermore, our study also showed that the lower the economic situation, the higher the prevalence of anxiety and depression. Since the included older adults all suffered from one or more chronic diseases that require long-term treatment and management, including medication, oxygen therapy, rehabilitation training, and other therapies, the cost of these treatments may impose a heavy financial burden on patients and families (Miao et al., 2022). What's more, because Zunyi City belongs to the hinterland of Southwest China and is relatively backward in terms of economic level, half of the elderly people it surveyed live in rural areas, and the elderly people mainly rely on the farming economy, with one crop a year, and the fact that the land isn't fertile, and their monthly incomes are low and unstable, which leads to extreme caution in the amount they spend on daily expenses. In contrast, the elderly in this study, all of whom have chronic diseases, require frequent medical treatment, and the accumulation of medical expenses may be beyond affordability, generating financial stress and anxiety, which may lead to symptoms of anxiety and depression. In addition, older adults with chronic diseases require long-term medication to maintain their health, and in this case, older adults have limited pensions or retirement funds, and the fear of depleting their savings or burdening family members with ongoing medical expenses may further lead to stress and depression.

Studies have shown that rural areas typically exhibit a severe shortage of mental health services compared to urban settings (Miao et al., 2022; Colon-Rivera and Dixon, 2020). As China's rapid urbanization has resulted in more young people leaving rural areas in search of employment opportunities, the proportion of older people living alone in rural areas has gradually increased. The gap between urban and rural areas in terms of income, social security system and infrastructure development has exacerbated the sense of loneliness among rural elderly living alone (Ren and Lu, 2021). As they are living alone, communication with the outside world is further reduced and they feel more isolated due to the loss of their social roles, and they feel more depressed or lonely compared to their relatives living with them and elderly people living in institutions (Hu et al., 2023), which can lead to the development of anxiety (Yu et al., 2020). In addition, studies have shown that living in an institution is a protective factor because there are more people of the same age in the institution who can interact with each other, the institution can provide a diversified model, there are professional caregivers who can provide around-the-clock detailed services and the nursing home can provide relatively convenient access to medical care and rehabilitation conditions (Li, 2024). This study also demonstrated that chronically ill older adults in rural areas have more fragile social support compared to urban populations, a finding supported by an Ecuadorian study (Madrid Miles et al., 2022). Since social support is one of the key factors in alleviating anxiety and depression symptoms and improving mental health, the lack of social support can cause a more pronounced mortality rate due to anxiety and depression symptoms (Rowland et al., 2023). Therefore, there is a need to enhance healthcare and social support for patients with anxiety and depression.

### 4.4 Physical activity, social activity, and ability to perform activities of daily living correlate with anxiety and depression

This study indicates that nearly half of the participants rarely or never engage in physical exercise or social activities, which is one of the risk factors for anxiety and depression among the elderly. A study has shown that participating in exercise, whether individually or in groups, can alleviate anxiety, and higher frequency of physical activity is associated with reduced levels of depression and anxiety (McMahon et al., 2017). Additionally, research suggests that regular participation in social activities helps avoid negative emotions and improves neurological function (Wang R. et al., 2019). Furthermore, a study highlights that chronic diseases in the elderly can lead to physical impairment and functional limitations, which may negatively impact their ability to perform daily living activities, potentially explaining why older adults are less likely to engage in physical exercise and social activities. However, in this study, mild dependency in daily living activities was identified as a protective factor. This is because mild dependency is more likely to draw attention and support from family members and others, and individuals with mild dependency can manage their lives without relying heavily on external assistance, making them less prone to experiencing symptoms of anxiety and depression.

### 4.5 Correlation between health status and anxiety and depression

The results of this study show that poor self-assessed health status is one of their risk factors. Self-assessed health status depends not only on the actual health status of patients with chronic diseases, but also on their perceived health status, which to a certain extent reflects older people's awareness of their own physiological and psychological conditions and changes, and those who self-assessed as fair or unhealthy tended to be lower than those who self-assessed as healthy in terms of their psychological status and social adjustment, and they were at increased risk of developing depression and anxiety (Fang et al., 2024). In addition, it has been shown that disturbed sleep quality increases the risk of reporting poor health, and self-reported rest/sleep deprivation appears to be a strong predictor of self-assessed health (Frange et al., 2014; Nordin et al., 2024). In our current multitude of studies, sleep disorders are one of the influencing factors that contribute to the occurrence of depression and anxiety in the elderly population. As anxiety and depression are usually accompanied by concerns and worries about falling asleep, it leads to the formation of a negative cycle of insomnia and anxiety-depression perpetuation (Fang et al., 2019). Meanwhile, some studies have shown a causal relationship between sleep disorders and debility (Lu et al., 2024). Debilitation leads to a significant increase in hospitalization rates, hospitalization costs, and morbidity and mortality rates, which seriously affects their quality of life, increases healthcare consumption, and aggravates the burden on families and society (Gong et al., 2022). Increased levels of debility are accompanied by increased levels of anxiety and depression (Hiriscau and Bodolea, 2019). In addition, with studies have shown that malnutrition can cause debilitation (Zhao et al., 2021). The bidirectional correlation between nutritional status, which is an important modifiable factor of frailty, which leads to malnutrition, and malnutrition, which leads to multi-systemic dysfunction and further aggravates frailty, creates a vicious circle in the elderly population. Therefore, regular attention to the physical health status of older persons is essential.

### 4.6 Limitations

First, only self-assessment tools were used to determine the presence of anxiety and depression, and no confirmation was made through diagnoses by psychiatrists. It is recommended that further research be conducted to gain a deeper understanding of the mental health of elderly patients with chronic diseases in Zunyi. Second, due to cultural, healthcare, and socioeconomic differences, the results may not be generalizable to other regions. Third, the cross-sectional design can only provide static data at a specific moment and cannot observe the changes in the prevalence of anxiety and depression and their influencing factors over time. Fourth, the cross-sectional design usually relies on self-reported data from respondents, which may be subject to recall bias or information bias. Future research should conduct longitudinal follow-up studies in multiple regions or institutions, analyze regional differences, and further explore the interaction mechanisms between chronic diseases, anxiety, and depression to provide scientific evidence for policy-making and data support for policy improvement.

## 5 Conclusion

The prevalence of anxiety among elderly individuals with chronic diseases in Zunyi City, China, is higher than the global average, while the prevalence of depression is lower than the global level. Patients with cardiovascular diseases exhibit higher rates of anxiety, depression, and the co-occurrence of both conditions. Living in rural areas, having no children, moderate to severe dependency in daily living activities, poor self-rated health status, malnutrition, frailty or pre-frailty, and sleep disorders are all independent risk factors for anxiety and depression among elderly individuals with chronic diseases. In view of this, under the leadership of the government, multiple parties such as communities and medical institutions should be united to actively carry out mental health education activities for the elderly with chronic diseases. Through diverse channels like community lectures, distribution of brochures, and online courses, knowledge about the symptoms, causes, and treatment methods of anxiety and depression should be widely popularized. Meanwhile, local governments and health departments need to increase investment in the construction of medical facilities in rural and remote areas of Zunyi, vigorously cultivate professional psychotherapy talents, and strive to enhance the primary medical institutions' capabilities in diagnosing and treating the elderly's mental problems. In addition, investment in mental health services should be increased, and specialized mental health service centers for the elderly should be established. Mental health services should be organically integrated with the management of chronic diseases in the elderly to build a comprehensive health management model, ensuring that elderly patients can receive mental health support while undergoing treatment for chronic diseases.

## Data Availability

The raw data supporting the conclusions of this article will be made available by the authors, without undue reservation.
